# Invasive aspergillosis complicated in a patient with non-small cell lung cancer harboring *RET* fusion during treatment with RET-TKIs: a case report and literature review

**DOI:** 10.3389/fonc.2024.1431908

**Published:** 2024-11-19

**Authors:** Kaidiriye Setiwalidi, Yimeng Li, Yuyan Ma, Zhanpeng Hao, Yujia Zhao, Yuxin Zhang, Xuan Liang, Tao Tian, Zhiping Ruan, Yu Yao, Xiao Fu

**Affiliations:** Department of Medical Oncology, The First Affiliated Hospital of Xi’an Jiaotong University, Xi’an, China

**Keywords:** NSCLC, *RET*, pralsetinib, selpercatinib, invasive aspergillosis

## Abstract

Pralsetinib and selpercatinib have been approved as specific tyrosine kinase inhibitors (TKIs) for the treatment of patients with non-small cell lung cancer (NSCLC) harboring rearranged during transfection (*RET*) fusion and mutation. However, adverse events associated with pralsetinib and selpercatinib are not fully understood, especially in the real world. In this case, invasive aspergillosis that appeared concurrent with RET-TKI targeted therapy is proposed to be an additional adverse drug reaction (ADR) that was not mentioned in previous reports. Here, we describe the process of clinical diagnosis and treatment of invasive aspergillosis and attempt to explore its possible pathogenesis in association with RET-TKI targeted therapy, with the aim of providing clinicians a more in-depth understanding of the ADR associated with RET-TKIs, as well as to prevent serious outcomes caused by reduction or discontinuation of antitumor therapy.

## Introduction

Lung cancer is the most commonly occurring cancer worldwide, with the highest mortality rate according to the data released by the World Health Organization (WHO) cancer agency in February 2024, and approximately 80%–90% of newly diagnosed lung cancers are non-small cell lung cancer (NSCLC) (1). With the rapid development of precision medicine, targeted therapies based on oncogenic driver genes have changed the treatment mode of NSCLC for more than a decade, especially for non-squamous cell carcinomas of NSCLC. Rearranged during transfection (*RET*) is an oncogenic driver gene located on the long arm of autosome 10 (10q11.2) and encodes a transmembrane glycoprotein receptor tyrosine kinase, which accounts for approximately 1%–2% in NSCLC (2). After interacting with ligands from the glial cell line-derived neurotrophic factor (GDNF) family, it forms a dimer and autophosphorylates on several tyrosine residues in a specific *RET* cytoplasmic domain (3, 4), ultimately resulting in the expansion of intracellular signaling and transcriptional pathways such as RAS/MAPK, JAK/STAT, and PI3K/AKT, which promote survival, proliferation, and migration (3, 5). Since its discovery, *RET* and its receptors have been implicated in a variety of cancers, including thyroid cancer, lung adenocarcinoma, colorectal cancer, and chronic monocytic leukemia (6, 7). Among them, the majority of *RET* fusion-positive NSCLC patients tend to be relatively young (<60 years of age), never smoked or lightly smoked, with the histological type of adenocarcinoma, with brain metastases at the initial diagnosis (5).

*RET* fusion-positive NSCLC has not benefited greatly from chemotherapy (8) or immune checkpoint inhibitors (ICIs) (9) although they have been widely used in patients with driver gene-negative NSCLC and achieved breakthrough progress. In the early phase of targeted therapy for *RET* fusion-positive NSCLC, the first-generation multikinase inhibitors (MKIs) with auxiliary *RET* activity such as sorafenib, cabozantinib, and vandetanib were commonly used. However, these MKIs were eliminated due to a lack of specificity, resulting in numerous off-target toxicities and a low objective response rate (ORR) (10). Thanks to its well tolerability, robust intracranial activity, and high specificity, the new RET-TKIs pralsetinib and selpercatinib have ushered in a new era of *RET* precision therapy. The U.S. Food and Drug Administration (FDA) approved pralsetinib and selpercatinib 3 years ago for the treatment of *RET* fusion-positive NSCLC. Adverse drug reactions (ADRs) are a significant factor influencing the therapeutic impact and course of patients receiving targeted therapy, and RET-TKIs are no exception. According to the updated data from the ARROW and LIBRETTO-001 trials, the most frequent ADRs of pralsetinib with a grade of 3 or higher were anemia (12%), hypertension (12%), and neutropenia (20%) (11), while the most common grade 3 or worse ADRs of selpercatinib were hypertension (13.2%), increased alanine aminotransferase level (9%), and increased aspartate aminotransferase level (6.3%) (12). However, as novel targeted drugs, the clinical efficacy and safety of RET-TKIs are still being explored in real-world practice. This study has been approved by the Ethics Committee of the First Affiliated Hospital of Xi’an Jiaotong University ( Ethics number: XJTU1AF2024LSYY-111).

## Case presentation

A 56-year-old man was admitted to our hospital in March 2019, with an incidental finding of a mass in the upper lobe of the right lung and elevation of tumor markers during a physical examination without any symptoms. He had a history of hypertension and colon polyp, without diabetes, coronary heart disease, and endemic mycosis exposure history. He did not smoke cigarettes or drink alcohol and had no family history of cancer or genetic disease. After exclusion of contraindications, the patient underwent thoracoscopic radical resection of the mass in the right upper lung lobe mass plus wedge resection of the right middle lung lobe on 21 March 2019 (intraoperative detection revealed that the mass was located in the anterior segment of the right upper lobe and invaded the right middle lobe). Postoperative pathological examination revealed adenocarcinoma (5 cm × 3 cm × 2 cm), with invasion of the visceral pleura, middle lobe of the right lung, and lymph node metastasis. According to the 8th American Joint Committee on Cancer Staging System (13), the patient’s postoperative staging was pT_4_N_1_M_0_, stage IIIA. Carboplatin and pemetrexed adjuvant chemotherapy every 21 days for four cycles and thoracic radiation therapy (TRT) were given 4 weeks after surgery.

**Figure 1:**
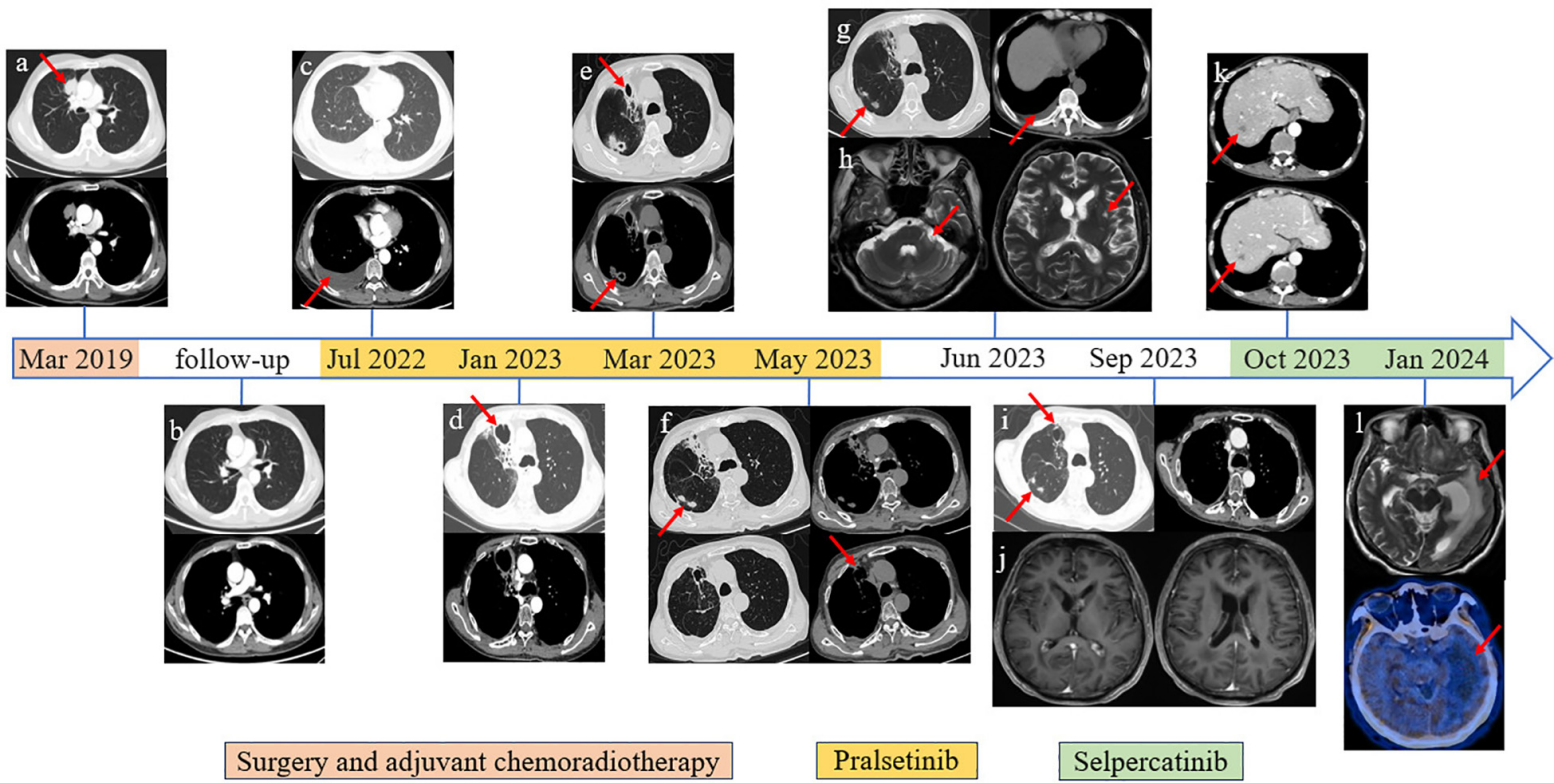
Imaging information throughout the disease course. **(A)** Primary lung tumor in the upper lobe of the right lung. **(B)** Follow-up chest CT scan image after surgery and adjuvant chemoradiotherapy. **(C)** Disease progression revealed by right pleural effusion cell smear. **(D)** Bronchial stenosis in the middle medial segment of the right lung, surrounding obstructive pneumonia and irregular cavity shadow, approximately 45 * 42 mm in size. **(E)** Cavity in the middle medial segment of the right lung was smaller than before, approximately 21 * 22 mm in size; newly added multiple irregular lesions with cavities. **(F)** The lesions on the middle medial segment of the right lung were unchanged. **(G)** The lesions were the same as above; recently presented right pleural effusion. **(H)** Abnormal enhancement in bilateral lateral ventricles and left cerebellar angle area. **(I)** The lesions on the middle medial segment of the right lung had shrunk. **(J)** The circularly enhancing lesion in bilateral lateral ventricles and left cerebellar angle area had shrunk. **(K)** Distant metastasis was discovered in the liver through the abdominal CT scan. **(L)** Cranial MRI revealed a new large edema on the left lateral ventricle compared with the previous examination, and further PET/CT (^18^F-FET) scan showed that there was no abnormal nuclide concentration in the edema area.

**Figure 2:**
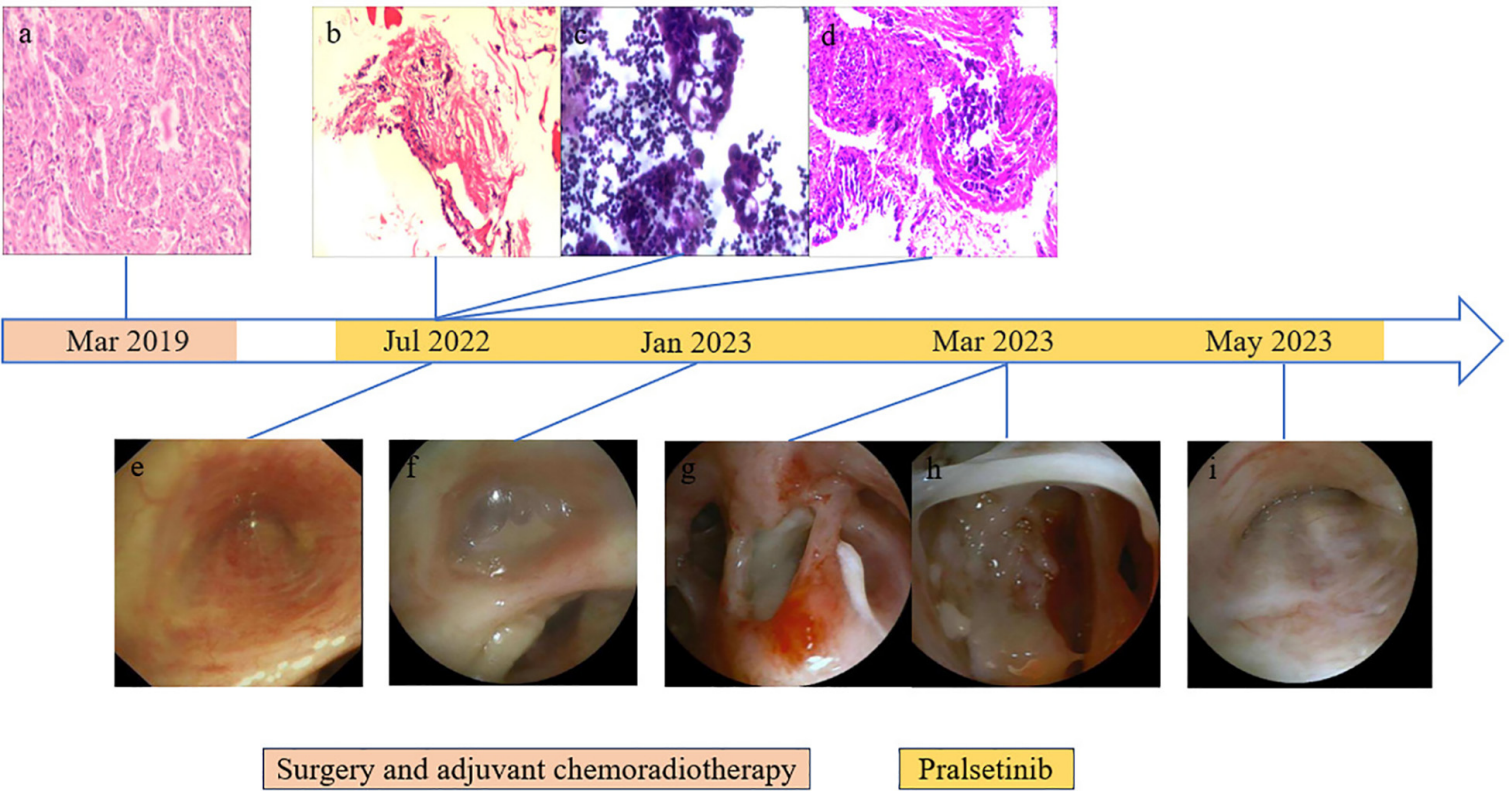
Results of endoscopic and histopathological examinations. **(A)** HE staining of surgical specimens showed lung adenocarcinoma. **(B)** Right pleural puncture pathology showed chronic inflammation of fibrous adipose tissue and a little skeletal muscle tissue. **(C)** On the right side, pleural effusion smear and sediment paraffin section found gland cancer cells; HE staining shows CK7(+), CEA(+), TTF-1(+), NapsinA (+), CDX2(−), CR(−), and D2-40(−). **(D)** Biopsy of lesions in the middle lobe of the right lung showed a small amount of poorly differentiated adenocarcinoma infiltration in the bronchial mucosa and a very small number of heterologous glandular epithelial cells in the cellulose exudation. HE staining indicated HER2(0), ALK(−), and PD-L1 (TPS: +1%). **(E)** Bronchoscopy showed mucosal eminence and lumen occlusion of the right middle lobe bronchus. **(F)** Right middle lobe bronchial tube lumen occluded with viscous discharge. **(G)** Excessive purulent discharge obstructing the lumen was seen in the right intermediate bronchus. **(H)** White camouflaged secretions can be seen in the dorsal segment of the lower lobe of the right lung covering the blocked lumen. **(I)** Bronchoscopy showed lumen occlusion of the right middle lobe bronchus.

After being discharged from the hospital, the patient received regular follow-up examinations and remained disease-free for 36 months. Unfortunately, in July 2022, disease progression was revealed by chest computed tomography (CT) scan, ultrasound bronchoscopy (EUBS) needle aspiration biopsy, and right pleural effusion cell smear (see **Figures 1**, **2** for details). The biopsy tissue was sent for next-generation sequencing (NGS) with a 14-gene lung cancer panel, and *KIF5B-RET* fusion was detected. Additionally, co-alterations were not identified for *EGFR*, *ALK*, *ROS1*, *MET*, *HER2*, *BRAF*, or *KRAS*. The Eastern Cooperative Oncology Group (ECOG) performance status score was 1. The patient was treated with pralsetinib, which was administered orally at a dose of 400 mg once daily since July 2022.

From January 2023 to May 2023, the patient went to the hospital several times due to a fever accompanied by cough and sputum. The test results of respiratory virus antibodies detection, atypical pathogen antibodies detection, COVID-19 nucleic acid test, sputum cytology, and sputum culture were all negative. The value of serum 1,3-β-d-glucan testing (G) and serum galactomannan testing (GM) increased. Chest CT scan showed multiple irregular cavities. *Pseudomonas aeruginosa*, *Aspergillus fumigatus*, *Candida albicans*, and *Pneumocystis jirovecii* were found in the bronchoalveolar lavage fluid, which was consistent with prior reports (14). During hospitalization, the patient received combined antimicrobial therapy with discontinuation of pralsetinib, his temperature returned to normal, and his symptoms such as cough and sputum were improved. After discharge from the hospital, he received voriconazole combined with a reduced pralsetinib dose of 200 mg once daily to control the primary disease, and intermittent fever occurred repeatedly (see **Supplementary Table 1** for details).

On June 2023, the patient visited the hospital once again due to dizziness with nausea, vomiting, and difficulty walking. Confusion and slurred speech, choking when drinking water and difficulty in swallowing, partial limb numbness, and meningeal irritation signs were not presented during the physical examination. The ECOG performance status score was 4. The chest CT scan revealed an additional pericardial and pleural cavity effusion. Cranial magnetic resonance imaging (MRI) showed abnormal enhancement in bilateral lateral ventricles and left cerebellar angle area, considering the possibility of central nervous system (CNS) infection. Lumbar puncture and NGS detection of cerebrospinal fluid proved CNS infection (see **Supplementary Tables 2**, **3** for details). He received antifungal therapy in conjunction with dehydrated cranial pressure, and pralsetinib was discontinued in June 2023. After being discharged from the hospital, the patient continued taking voriconazole for 12 weeks, and the follow-up chest CT scan and brain MRI showed lesions to be decreasing. Due to the fact that the patient suffered from recurrent infections, which significantly impacted his physical wellbeing, and the discontinuation of pralsetinib led to the elevation of tumor markers, the decision was made to transition to selpercatinib at a dose of 160 mg twice daily from October 2023.

**Figure 3:**
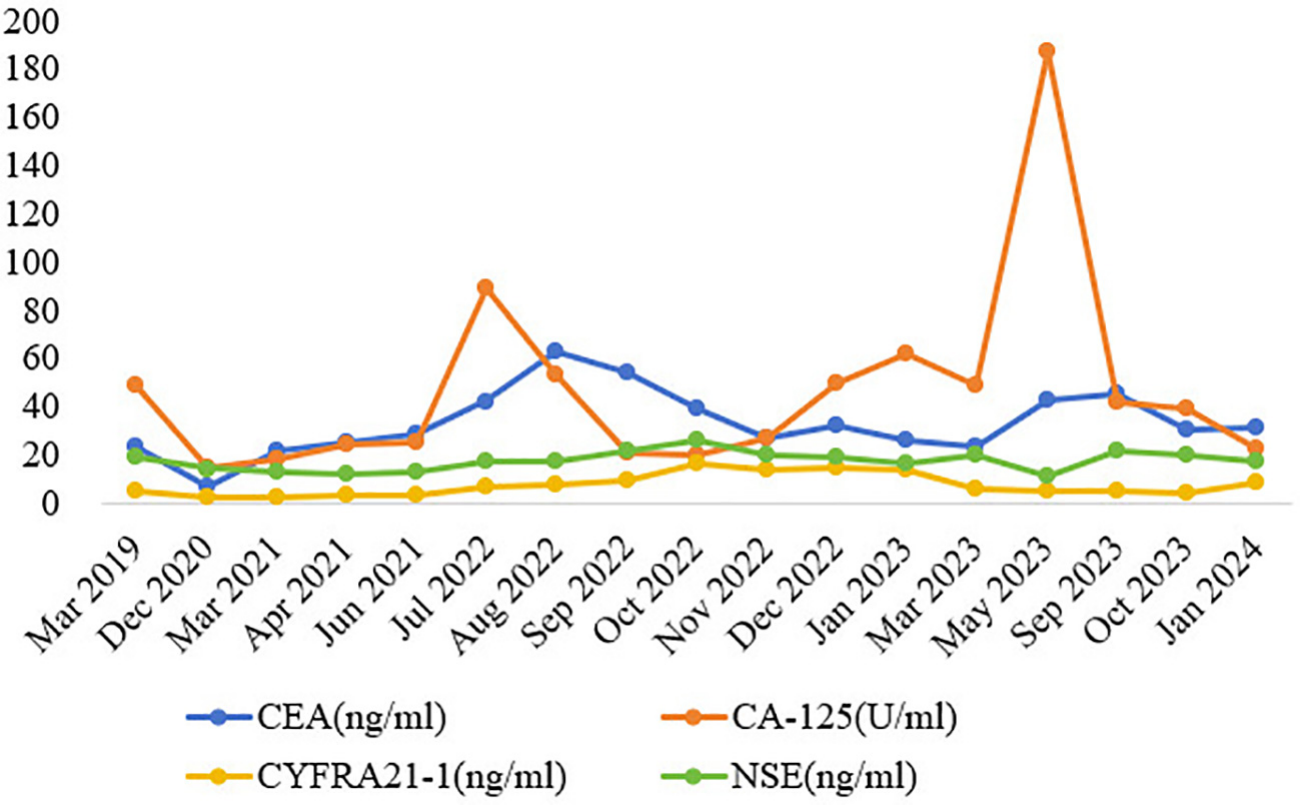
Dynamic monitoring of tumor markers during treatment. Note: CEA, carcinoembryonic antigen; CA-125, carbohydrate antigen 125; CYFRA21-1, cytokeratin 19 fragment; NSE, neuron-specific enolase.

Follow-up CT scan and tumor markers showed that the tumor did not progress, but dizziness accompanied by limb weakness appeared again in January 2024. The patient’s cranial MRI revealed a new large edema on the left lateral ventricle compared with the previous examination, and further PET/CT (^18^F-FET) scan showed that there was no abnormal nuclide concentration in the edema area, which was not consistent with neoplastic changes, as shown in **Figures 1**, **3**. Combined with the previous history of intracranial *Aspergillus fumigatus* infection, consideration of the intracranial lesions as recurrent CNS infections was not excluded. The patient and his family requested discharge, and since then, the patient’s general condition deteriorated and he died in March 2024.

## Discussion

Pralsetinib and selpercatinib are potent and highly selective RET-TKIs that target *RET* alterations, including fusion and mutation, irrespective of the tissue of origin. Recently, the results from clinical trials demonstrated significant clinical benefits and tolerable toxicity of pralsetinib and selpercatinib, respectively. According to the updated data from the ARROW and LIBRETTO-001 trials, pralsetinib had an ORR of 72% for treatment-naive patients and 59% for patients with prior platinum-based chemotherapy, and the median progression-free survival (mPFS) rates were 13.0 and 16.5 months, respectively (11), while selpercatinib had an ORR of 84% for treatment-naive patients and 61% for patients with prior platinum-based chemotherapy, and the mPFS rates were 22.0 and 24.9 months, respectively (12).

This patient is a middle-aged man with *RET* fusion-positive NSCLC, without a history of chronic respiratory disease or smoking. A regular review of the chest CT scans showed no pulmonary infectious lesions. RET-TKIs provided significant benefits to patients, and a decline in tumor markers was observed, which is consistent with previous studies. At the same time, we observed an effect not previously documented in clinical trials. After 5 months of antitumor treatment with pralsetinib, the patient developed invasive pulmonary aspergillosis, and symptoms occurred repeatedly even after the reduction or discontinuation of pralsetinib, ultimately progressing to CNS infection. In light of the ADR of pralsetinib, the antitumor therapy was discontinued, and a powerful anti-infective therapy was initiated, which then controlled the infection. However, after 2 months of antitumor treatment with selpercatinib, large edema of the left lateral ventricle was detected without abnormal nuclide concentration in PET/CT (^18^F-FET), suggesting a correlation between the adverse reactions and the targeted therapy drug. Moreover, the patient in this case had been regularly taking nifedipine controlled-release tablets to control blood pressure for many years, and the patient experienced no discomfort in the past. Furthermore, there was no record of adverse reactions to infection in its instructions. Therefore, the possibility of nifedipine controlled-release tablets causing invasive aspergillosis was not considered. In addition, the patient had no history of diabetes or long-term use of broad-spectrum antibiotics, glucocorticoids, or immunosuppressants during the treatment. Therefore, we concluded that the occurrence of invasive aspergillosis may be related to RET-TKIs.

We reviewed the literature on infections that occurred after treatment with RET-TKIs and identified six studies published locally and abroad, as shown in **Table 1**. Most of the reported opportunistic infections were associated with pralsetinib, whereas no association was reported with selpercatinib. Of these, *Mycobacterium tuberculosis* (MTB) infections were the most common, including pulmonary and extrapulmonary tuberculosis, and the average time to infection was approximately 3 months after starting RET-TKI therapy. In view of the above data, it is suggested that opportunistic infectious events may be a significant safety issue for patients who are receiving RET-TKIs. As reported in the updated data from the ARROW and LIBRETTO-001 trials, 26.5% and 4% of the patients treated with pralsetinib and selpercatinib developed pneumonia, respectively, involving multiple pathogens, including a variety of bacteria, cytomegalovirus, and influenza virus, but no fungal infection events had been recorded after treatment with pralsetinib or selpercatinib (11, 12). It is important to note that clinical trials typically enroll carefully selected patients with fewer comorbid conditions and immunosuppressive conditions than average patients, and there may be a higher incidence of opportunistic infection in the real world.

**Table 1:** Summary table of published literature on infections occurred after treatment with RET-TKIs.

Patient No.	Sex	Age	Primary cancer	Stage	Type of gene alternation	Type of RET-TKI	Pathogen	Site of infection	Time interval from starting RET-TKIs to infection	Reference
1	F	58	NSCLC	NA	*RET* mutation	Pralsetinib	*Herbaspirillum*	Sepsis	NA	(15)
2	F	57	NSCLC	III	*RET* fusion- positive	Pralsetinib	MTB	Neck lymph node	2.0 months	(16)
3	F	51	NSCLC	IV	*RET* fusion- positive	Praisetinib	MTB	Neck lymph node, peritoneum, liver, spleen	3.5 months	(16)
4	M	81	NSCLC	IB	*RET* fusion- positive	Pralsetinib	*Cryptococcus*; MSSA	Pulmonary	1 month	(17)
5	M	44	MTC	IV	*RET* mutation	Pralsetinib	MTB	Pulmonary, pleura	6 months	(18)
6	M	62	PTC	IV	*RET* fusion- positive	Pralsetinib	MTB	Bronchi, Mediastinal and hilar lymph nodes	9 months	(19)

F, female; M, male; NSCLC, non-small cell lung cancer; MTC, medullary thyroid cancer; PTC, papillary thyroid carcinoma; NA, not available; MTB, *Mycobacterium tuberculosis*; MSSA, methicillin-sensitive *Staphylococcus aureus*.

The mechanism of RET-TKIs in causing invasive aspergillosis is not well understood, and there are no clinical cases reported to date, as the drugs have only recently entered the market. Although pralsetinib and selpercatinib are highly selective against RET kinases, they have also been shown to inhibit some non-RET kinases, such as JAK 1/2 and VEGFR (20). The JAK-mediated intracellular signaling pathway plays a key role in immune regulation and host defense (21). Treating autoimmune diseases or cancer with JAK inhibitors has been well documented to be associated with an increased frequency of infections, including fungal and mycobacterial infections and reactivation of shingles and viral hepatitis B (22–24). Therefore, RET-TKIs may cause invasive aspergillosis due to their off-target effect on JAK 1/2. In addition, the antifungal drugs voriconazole and fluconazole used during infections act as CYP3A inhibitors, resulting in increased exposure to RET-TKIs, which may be attributed to recurrent infection events.

However, there are several factors that may influence our conclusion. A previous study found that lower body mass index (BMI), underlying interstitial lung disease, and a history of lung cancer surgery or radiation pneumonia were identified as risk factors for pulmonary infections (25). Nevertheless, Meng’s research systematically reviewed existing publications on NSCLC treated with radiotherapy and TKIs, and it suggested an acceptable risk of severe treatment-related pneumonitis and rare mortality in patients with NSCLC (26). In brief, further prospective studies with a larger number of patients and a longer-term follow-up are warranted to support our conclusion that RET-TKIs may cause invasive aspergillosis.

Infection during antitumor therapy will not only prolong a patient’s hospitalization time but also increase hospitalization expenses, and even in severe cases, the infection quickly spreads to other organs and often leads to death. Additionally, it will lead to a dose reduction of antitumor drugs, delay of time, and even suspension of treatment, which will affect the antitumor efficacy and the patient’s survival time. Therefore, it is crucial to manage infection events during antitumor therapy. In this case, the patient’s symptoms improved after pausing pralsetinib and receiving a powerful anti-infective therapy. To avoid affecting the timing of antitumor treatment and in consideration of drug safety, the antitumor treatment regimens were replaced by another RET-TKI—selpercatinib—after the infection was absolutely controlled. However, selpercatinib caused a new large edema of the left lateral ventricle, and the patient’s general condition deteriorated since then, and he died in a few months. In conclusion, this case describes real-world experiences of using RET-TKIs in patients with *RET* fusion-positive NSCLC, underscoring the importance of adequate baseline assessment and ongoing monitoring of immune function, infection biomarkers, and chest CT scans to inform future clinical practice. If there is a fever or respiratory symptoms that cannot be explained by the primary disease, it is important to consider ADRs.

As mentioned above, *RET* fusion-positive NSCLC patients are prone to brain metastases. A single-center *RET* retrospective study from South Korea analyzed 59 treated *RET* fusion-positive NSCLC patients, and the results showed that a total of 17 patients (28.8%) had a brain lesion at the initial diagnosis, while 11 additional patients (18.6%) developed brain metastases during follow-up (27). Therefore, when *RET* fusion-positive NSCLC patients have single or multiple lesions on brain MRI, it is necessary to distinguish them from the primary tumor with brain metastases, which depends on medical history, clinical manifestations, and appropriate imaging examinations. CT is limited in the differential diagnosis of brain abscess and brain metastases, while MRI-enhanced scan is more sensitive than CT or MRI plain scan, and it is easier to detect more and smaller lesions at the early stage. In addition, diffusion-weighted imaging (DWI) and PET/CT provide valuable evidence for the differential diagnosis of benign and malignant diseases of the CNS, which can help identify lesion properties and avoid patients undergoing unnecessary invasive procedures such as puncture or craniotomy. Clinicians should improve their understanding of CNS infection when treating tumor patients and avoid misdiagnosing CNS infection lesions as the progression of tumor brain metastases.

## Conclusion

Pralsetinib and selpercatinib have been on the market for a short time in China. Although they have achieved remarkable efficacy in the treatment of *RET* fusion-positive NSCLC, it is still necessary to pay attention to the ADRs. This article presents a rare case of invasive aspergillosis induced by RET-TKIs in the treatment of *RET* fusion-positive NSCLC, suggesting that clinicians should pay attention to identifying ADRs, opportunistic infections, and tumor progression throughout the antitumor targeted therapy so as not to delay antitumor treatment.

## Data Availability

The original contributions presented in the study are included in the article/**Supplementary Material**. Further inquiries can be directed to the corresponding authors.
